# Role of 3D Volumetric and Perfusion Imaging for Detecting Early Changes in Pancreatic Adenocarcinoma

**DOI:** 10.3389/fonc.2021.678617

**Published:** 2021-09-08

**Authors:** Syed Rahmanuddin, Ronald Korn, Derek Cridebring, Erkut Borazanci, Jordyn Brase, William Boswell, Asma Jamil, Wenli Cai, Aqsa Sabir, Pejman Motarjem, Eugene Koay, Anirban Mitra, Ajay Goel, Joyce Ho, Vincent Chung, Daniel D. Von Hoff

**Affiliations:** ^1^National Medical Center & Beckman Research Institute, City of Hope Comprehensive Cancer Center, Duarte, CA, United States; ^2^Virginia G Piper Cancer Center, Honor Health, Scottsdale, AZ, United States; ^3^Molecular Medicine Division, Translational Genomics Research Institute (TGEN), Phoenix, AZ, United States; ^4^Department of Radiology, Massachusetts General Hospital, Boston, MA, United States; ^5^Department of Radiation Oncology, University of Texas MD Anderson Cancer Center, Houston, TX, United States; ^6^Department of Pathology, University of Texas MD Anderson Cancer Center, Houston, TX, United States; ^7^Molecular Diagnostic and Experimental Therapeutics, City of Hope Comprehensive Cancer Center, Monrovia, CA, United States

**Keywords:** 3D volumetric analysis, time intensity-density curve, perfusion analysis, pancreatic ductal adenocarcinoma, CT images

## Abstract

**Purpose:**

There is a major shortage of reliable early detection methods for pancreatic cancer in high-risk groups. The focus of this preliminary study was to use Time Intensity-Density Curve (TIDC) and Marley Equation analyses, in conjunction with 3D volumetric and perfusion imaging to demonstrate their potential as imaging biomarkers to assist in the early detection of Pancreatic Ductal Adenocarcinoma (PDAC).

**Experimental Designs:**

A quantitative retrospective and prospective study was done by analyzing multi-phase Computed Tomography (CT) images of 28 patients undergoing treatment at different stages of pancreatic adenocarcinoma using advanced 3D imaging software to identify the perfusion and radio density of tumors.

**Results:**

TIDC and the Marley Equation proved useful in quantifying tumor aggressiveness. Perfusion delays in the venous phase can be linked to Vascular Endothelial Growth Factor (VEGF)-related activity which represents the active part of the tumor. 3D volume analysis of the multiphase CT scan of the patient showed clear changes in arterial and venous perfusion indicating the aggressive state of the tumor.

**Conclusion:**

TIDC and 3D volumetric analysis can play a significant role in defining the response of the tumor to treatment and identifying early-stage aggressiveness.

## Introduction

Pancreatic cancer is one of the leading causes of cancer death globally (1). Due to conventional treatments having a minute impact on its natural progression, this disease is considered a major unresolved health problem. Metastases commonly develop in patients with pancreatic cancer resulting in high mortality (2). The average 5-year survival rate is less than 5% and death can occur to most patients 6 months after cancer diagnosis (1, 3). The incidence of pancreatic cancer is higher in developed countries due, in part, to the differences in diet and lifestyle (1). Risk factors for pancreatic cancer include smoking cigarettes, a family history of pancreatic cancer, diabetes mellitus (Type I or Type II), obesity, consumption of a diet high in fat, alcohol use, and a lifestyle categorized by physical inactivity (4). One of the factors that contribute to the high rate of mortality stems from the nonspecific nature of early presenting symptoms. When the symptoms become severe enough for the individual to seek treatment, 80%–85% of patients present with the advanced unresectable form of this cancer (4). Although these symptoms are not specific and difficult to control, screening methods to identify the earlier stages of cancer can be addressed. Our primary focus for this preliminary study was to identify an approach that used the Time Intensity-Density Curve (TIDC) graphs to show its application in the early detection of pancreatic cancer. There are different types of pancreatic malignancies with Pancreatic Ductal Adenocarcinoma (PDAC) being the most common and severe, accounting for over 85% (4, 5). The cure involves a complete removal of the tumor through surgery; however only 10%–20% of PDAC patients are surgically eligible at the time of diagnosis (4).To compound this problem further, there is a lack of effective early detection tools for pancreatic cancer in high-risk populations (those with greater than 5% lifetime risk of pancreatic cancer) (4). Due to the lack of available early detection screening methods for pancreatic cancer, the fatality rates are greater, and the life expectancies from the time of diagnosis are lower as compared to other types of cancer (5, 6). Early screening methods for PDAC include Endoscopic Ultrasound (EUS) and Magnetic Resonance Imaging (MRI), which have not demonstrated improvement in the long-term survival (7) of patients. Despite the inability to enhance the long-term survival rates overall, these efforts in screening have paved the way for research in the creation of other modalities to detect pancreatic cancer. One of the current modalities is Computed Tomography (CT). CT exams are more accurate and robust in defining cancer characteristics while being the most common imaging modality available compared to EUS and MRI (7). **Table 1** provides a list of current diagnostic modalities and their respective advantages and limitations.

**Table 1:** Benefits and limitations of pancreatic cancer diagnostic modalities ^i, ii^.

Diagnostic modalities	Advantages	Limitations
**MDCT** **89% sensitivity** **90% specificity**	Most commonly available Best validated Cheapest	Nephrotoxicity Radiation exposure
**MRI** **89% sensitivity** **89% specificity**	Superior imaging Depicts local pancreatic disease Iodine-free and no radiation	Expensive Less available Contraindicated with some metal implants
**EUS +/- FNA** **85% sensitivity^iii^** **96% specificity^iii^**	Safe and less invasive High sensitivity Able to detect small lesions Able to take histological sample	Less available in some countries Operator dependent Inability to detect distant metastasis

i Adapted from Zhang L et al., World Journal of Gastroenterology. 2018; 24:2047-2060.^6^

^ii^CT, Computed tomography; MDCT, Multi-detector computed tomography; MRI, Magnetic resonance imaging; EUS, Endoscopic ultrasound; FNA, Fine needle aspiration; PET, Positron emission tomography.

^iii^Pooled sensitivity and specificity for EUS with FNA.

The International Cancer of the Pancreas (CAPS) Consortium recommends individuals who fall into a high-risk group to get screened for cancer beginning at age 50 using EUS or MRI, with annual surveillance if no pancreatic lesions are identified on baseline assessment (4, 8). Both modalities are sensitive and specific for detecting small lesions or early cancer without the risks of radiation exposure. However, EUS performs better in cases of small solid lesions and provides an opportunity for tissue diagnosis by FNA, while MRI Cholangiopancreatography (MRCP) with and without contrast is preferred for cystic lesions and often used as a secondary form of screening in cases where there is a high clinical suspicion for pancreatic cancer despite a negative CT (7).

This study evaluated the use of 3D quantitative volumetric and TIDC analysis for detecting PDAC characteristics. We believed that the switch from 2D to 3D imaging would negate some of the limitations identified with 2D imaging. Multi-phase multi-detector computed tomography (MDCT) is the first line modality of choice for the diagnosis of pancreatic cancer (9). These tests must be tailored to the location of the area to be screened and use the available tools to complete the process, which can be difficult Pancreas-specific screening and diagnostic protocols involve the use of images in the pre-contrast, early arterial (CT angiography), pancreatic, and portal venous phases. The early arterial phase is useful in delineating the aorta and superior mesenteric artery and the portal venous phase can assess venous involvement and liver metastases. There is conflicting data from researchers regarding the optimal phase for enhancement of pancreatic lesions. Some studies suggest a better visualization of PDAC as low-attenuation lesions during the arterial phase due to its hypo-vascularity (4), whereas others demonstrated better pancreatic enhancement and attenuation differences between the normal pancreas and tumor in the portal venous phase (4).

Pancreatic tumors are often hypo-vascular and ill-defined, with irregular texture and abnormal morphology (10). To better understand the long-term outcomes and prognostic factors associated with PDAC, multi-phase CT images of patients undergoing treatment for PDAC were analyzed using advanced 3D imaging software to characterize the perfusion and radio density of tumors at various stages. Our goals were to identify imaging biomarkers to help detect pancreatic cancer early and define features of pancreatic cancer that correlate with disease severity and treatment response. The findings from this study might help define the mechanisms and growth patterns of PDAC while laying the groundwork for further research in early PDAC detection.

## Materials and Methods

### Patient Population

A quantitative, retrospective, and prospective study was done using 41 adult patients with unresectable treatment naïve PDAC who were either enrolled in a clinical trial at Honor Health Cancer Center (Phoenix, AZ) or treated at City of Hope Comprehensive Cancer Center (Duarte, CA) as Standard of Care (SOC) non-study patients between 2015 and 2019.Consent was obtained from all clinical trial patients for the use of their images gathered from medical records for imaging analysis. As a result, there was limited control over the way the images were taken or its time frames. All patients were over the age of 18 and had been diagnosed with PDAC. We divided the participant sample into two groups, labeled Cohort 1 and Cohort 2.

Cohort 1 consisted of 22 patients enrolled in the clinical trial treated at Honor Health Cancer Center. Patients within this sample group received neo-adjuvant therapy (paricalcitol, paclitaxel protein bound, cisplatin, and gemcitabine) for up to 6 months with standard of care CT multiphase imaging. The original purpose for the Honor Health clinical trial was to see the resectability and CA 19-9 status within non-metastatic pancreatic cancer but we decided to use the preliminary imaging findings in this ongoing study and assess the volumetric response in Cohort 1 as well as Cohort 2. Patients with a positive metastatic disease were excluded from the study. Furthermore, patients whose tumors were deemed resectable after the completion of neo-adjuvant therapy were likewise removed from the study to receive SOC treatment due to changes in their condition.

Cohort 2 consisted of 19 patients treated at the City of Hope Comprehensive Cancer Center. Patients within this sample group received SOC therapy only and the CT multiphase imaging exam was performed at least every 3 months as a follow-up scan. Like Cohort 1, neo-adjuvant rules were not applied since most images were obtained retrospectively. Cohort 2 was divided based on the location where participants received care to ensure that variations in treatment, tools, or other variables did not affect the participants within their given groups. To make sure that there was uniformity in the participant samples (i.e., each patient in a participant group was exposed to similar variables as the other patients within the same participant group), we worked to increase the overall reliability of the data collected during the image acquisition process.

We submitted the complete requests for information in conjunction with the Health Insurance Portability and Accountability Act (HIPPA) authorization to obtain the records of all participants from the Honor Health and City of Hope Medical Records Departments. Following the receipt of the images, we then analyzed the scans to ensure that the images fit the necessary criteria. However, since we had no control over the image collection process, most of the data obtained could not be used due to issues with the imaging protocol. Additional exclusion criteria for these images included poor image quality, variations in time between images (e.g., too far apart or non-comparable to others within the sample set), and the use of non-standard practices in image collection. These variations, especially the latter, occurred more frequently than we anticipated, leaving the image sample size for TIDC at 28 patients out of the anticipated 41 participants from which scans were requested.

### Image Acquisition

For the PDAC cohorts, we required multiphase CT images for collection at Honor Health and City of Hope. All images from Honor Health and City of Hope used the same standard protocol to acquire the participant CT scans for analysis. First, the images were obtained from the imaging conducted with multi-phase CT. Most of the acquired images were scanned on a GE scanner with a standard pancreatic protocol which included pre-injection, arterial, and portal venous phases (11). Second, to maximize the coherency and accuracy of the analysis, the images must have used the standard iodinated contrast agent Isovue 370. Patient files from both cohorts indicated that each participant received a dose of 150 cc + 40 cc of saline that was injected at a rate of 5 cc per second into the antecubital vein of the participant.

For scanning parameters, the areas of interest were defined as the region above the diaphragm to the inferior liver margin for arterial phase images. The region from above the diaphragm to the superior aspect of the iliac crest was used for both pre-contrast and venous phase images. Multi-phase scanning parameter used in the image collection process was at 120 kV with a 0.5 second rotation time. All images were reconstructed as follows: pre-contrast phase images were reconstructed into 3-mm slices and images from arterial or venous phases into 2-mm slices. Images were reviewed by experienced radiologists to ensure that the image quality was sufficient but 3D Qi has some high-resolution issue which can be fixed in future upgrades. Of the 28 participant images obtained, 24 were of ample quality for analysis. Initial images were reconstructed into 0.625 x 1.25 mm-slices for 3D post-processing and uploaded from the Picture Archiving and Communication System (PACS) to advanced imaging software (GE Advantage Workstation 3.2) for volumetric analysis.

### 3D Volumetric Analysis

**Figure 1:**
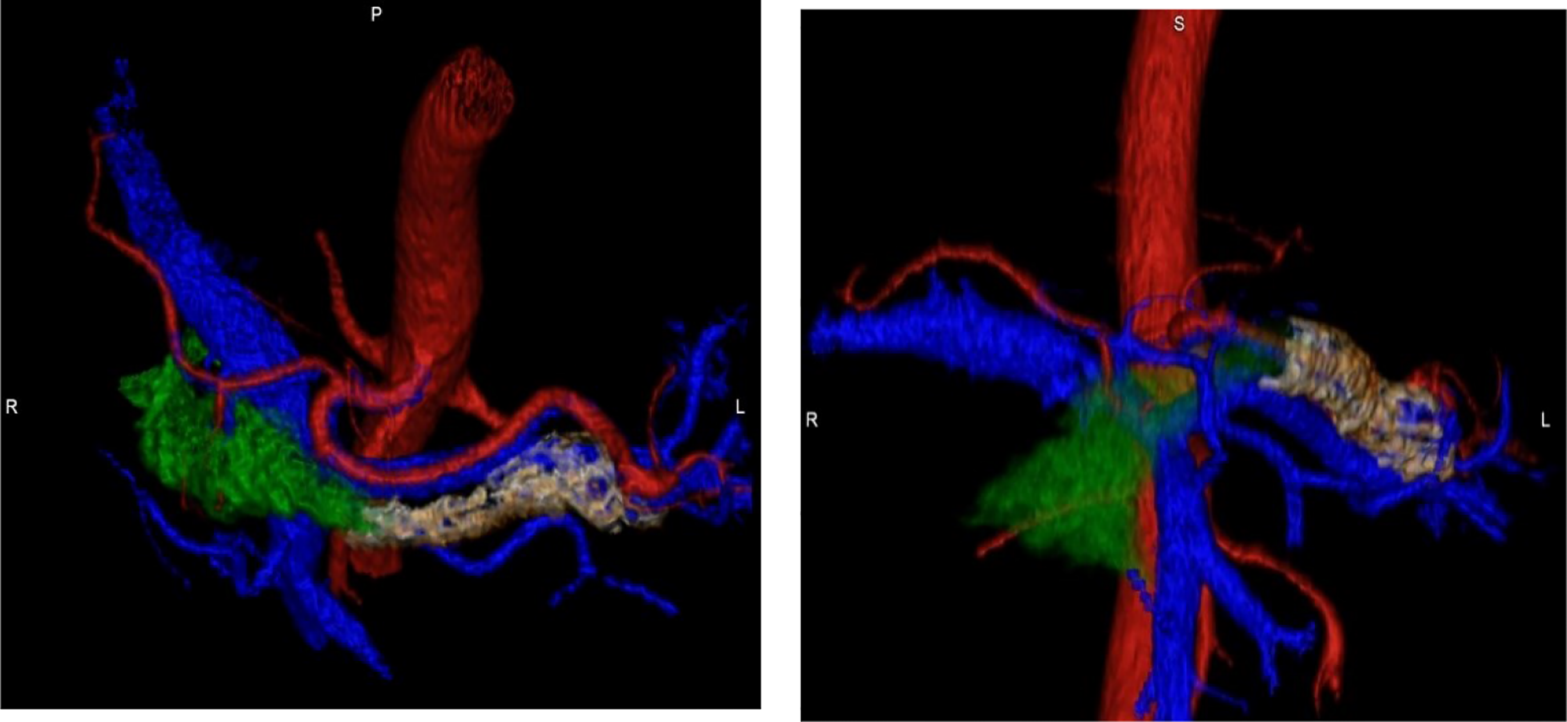
Pancreatic Tumor Volumetric Mapping.

A 3D imaging software application called GE AW 3.2 USA was used on the 28 PDAC patient CT images to conduct the volumetric analyses. This method of analysis refers to the total amount of a given substance, in this case, the pancreatic tumor, which was determined by measuring the volume that the tumor occupied (12–14). Similar analytical approaches had promising outcomes when exploring various medical conditions involving the pancreas and liver, providing adequate justification for its use in the context of this study (12–17). To confirm the accuracy of this method, multiple series were registered and regions of interest were identified prior to receiving the requested study images to define volumetric tumor quantification. Edits to the regions of interest were performed when needed to ensure that volumetric analysis was as accurate as possible using the available tools (see **Figure 1**).

**Figure 2:**
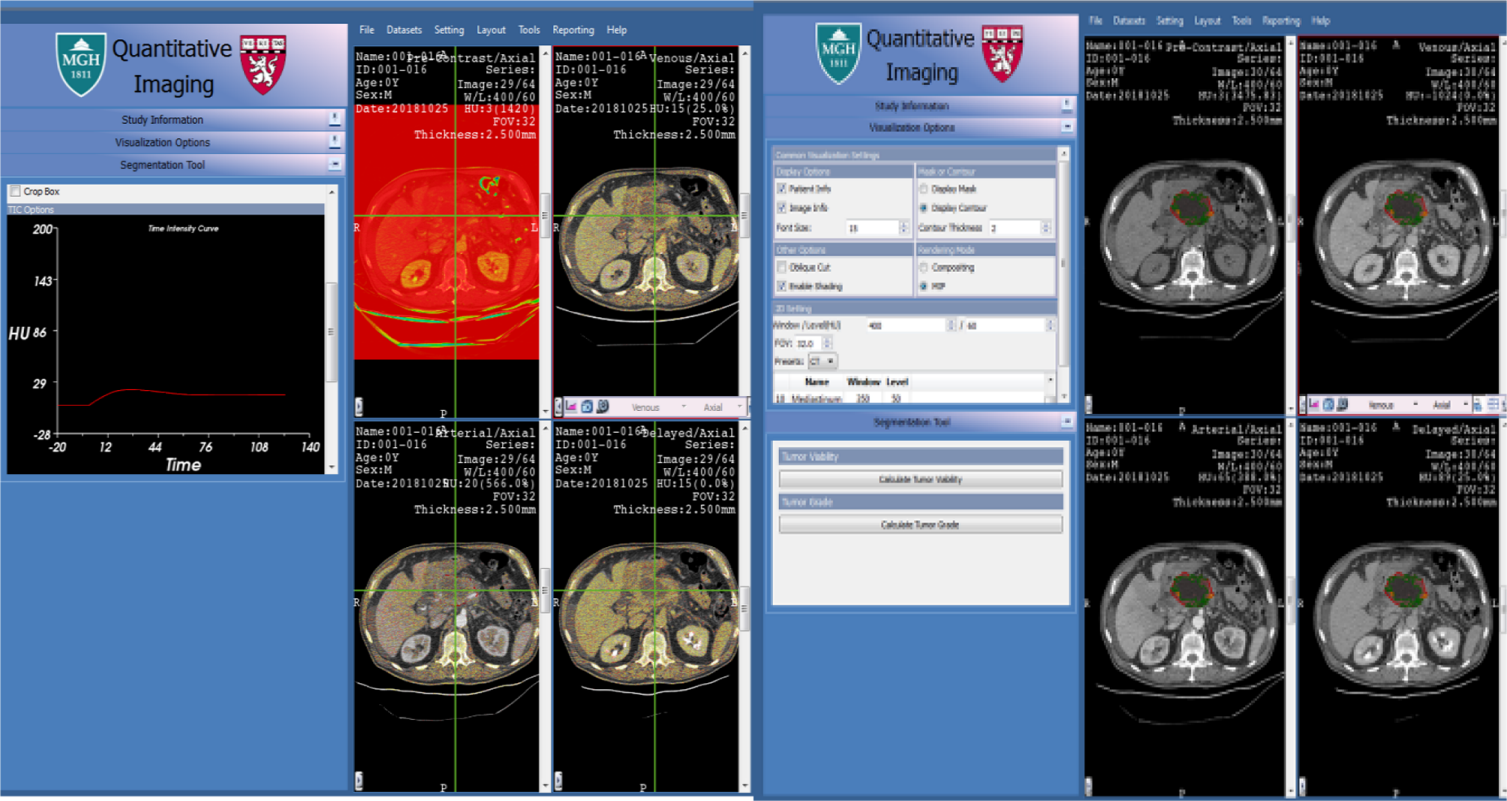
3D Qi Software.

Images were imported to the Massachusetts General Hospital application called 3D Quantitative Imaging software (3DQI). The PDAC tumor was identified by a radiologist and radiology expert. All series were opened side by side to do a comparison for better visibility and quantitative assessment (See **Figure 2**). Elaboration on the perfusion imaging characteristics on multiphase CT was a major goal of this research. The standard CT protocol for the PDAC imaging requires non-contrast with arterial and venous phase imaging. This preliminary study was designed to observe changes occurring during the wash-in and wash-out rates seen in perfusion imaging. This application has been used successfully in hepatocellular carcinoma (HCC) (18–21). HCC shows a complete wash-out in the venous and late phase imaging but the PDAC tumor becomes more vascularized in the venous phase which indicates missing information that might be critical to how PDAC is defined.

### Marley Equation

**Figure 3:**
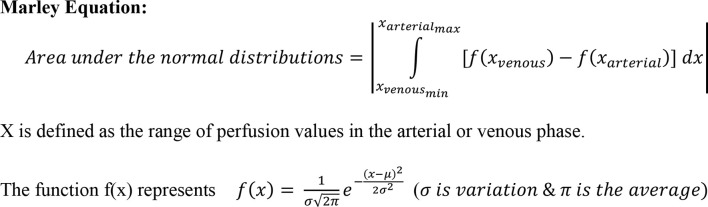
The Marley Equation.

The Marley equation was created and first investigated in the Rahmanuddin lab to describe the difference in the enhancement patterns between the arterial and venous phases (see **Figure 3**). This equation uniquely defines the characteristics of each pixel and flow inside the tumor which is aggressive in nature and can potentially be useable in the early detection of pancreatic cancer. In comparison to HCC imaging, PDAC has unique characteristics which are seen in the late arterial or venous phase imaging. It is visible in the venous phase which indicates the prolonged time of tumor vascularization. The longer the venous phase wash-out rate, the higher the tumor aggression. Marley equation helps to define the gap between the arterial and venous phases which can estimate the accurate change in the time on perfusion imaging. Our previous preliminary results showed that using the Marley Equation can be beneficial in observing the differences more precisely. This equation might be helpful in relating imaging perfusion to Vascular Endothelial Growth Factors (VEGF). **Figure 3** provides a breakdown of the Marley equation, indicating how it was used in the assessment within the current study.

### Time-Intensity-Density Curves

The potential in the application of TIDC, as a means of detecting tumor aggressiveness was seen using 3DQI software. Time-Density Curves for CT scans and Time-Intensity Curves for MRI scans provide a visual tool for the enhancement or reduction of a particular mass/lesion over time based on the intensity and density (22–25). Previous studies have demonstrated the utility of TIDC analysis in differentiating solid lesions in the pancreas using contrast-enhanced EUS (16), a first line diagnostic test (26–29). However, due to the limitations presented in the US examinations, compared to the test environment setting, and the associated concerns regarding accuracy across the application throughout treatment facilities in the United States, past researchers have noted the difficulties associated with standardizing these markers as a means of creating a unified evidence-based practice (EBP) for its use as a diagnostic modality (30–34).

Our focus was to identify an approach that used the TIDC to show how it can be applied in the early detection of pancreatic cancer. The identification of the TIDC patterns that correlate with PDAC responses to treatment would enable clinicians to better evaluate the severity of the disease, in addition to serving as a diagnostic screening modality. The TIDC model generated for the current study displayed information based on each pixel present in the multi-phase CT imaging within the tumors and tissues of interest in each participant. Through the integration of a computer-based software for pixel-by-pixel analysis, we were able to define the time-in and time-out quantitative numbers with a greater level of precision compared to past efforts (35–38). The artificial intelligence tool used was uniquely designed to differentiate the pixel-based change in contrast enhancement related to perfusion in multiphase imaging.

## Results

The Marley equation has proven that it is applicable in quantifying tumor aggressiveness. Using this equation, we were able to generate normal distribution histograms based on the minimum, maximum, and average tumor radio density, as measured in Hounsfield Units (HU). Tissue radio density refers to the opacity of the tissue as measured by how readily the different types of electromagnetic radiation passes through the tissue itself. HU is a common scale used in assessing CT scans and refers to a linear transformation of the radio density of various materials. The common HU reference point is distilled water at a standard pressure and room temperature set as zero. The tissue radio density on CT images, measured in HU, changed with respect to time. Specifically, analysis showed that the difference in tumor perfusion during the arterial and venous phases increases with respect to time. This change continuously increases during the later stages of the venous phase without changing the tumor dynamic contrast enhancement.

**Figure 4:**
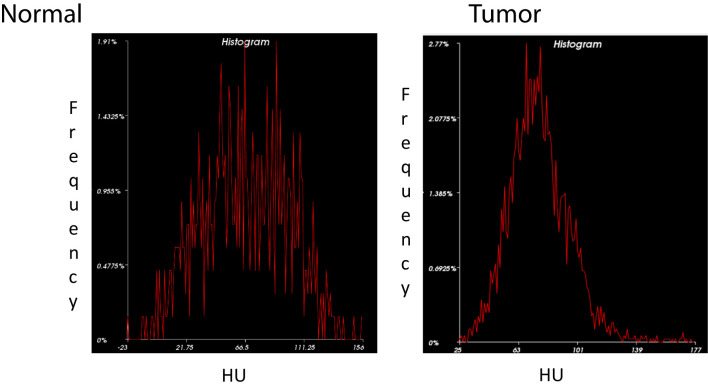
Histogram Analysis of Normal Pancreas *vs*. PDAC. Histogram distribution of the normal pancreas *(left)* shows greater variation in density representative of its heterogeneous echotexture compared to a more homogenous pattern in PDAC *(right)*.

Our preliminary study highlighted the following major areas based on perfusion imaging. The Marley equation showed that perfusion changes play a major role in tumor kinetics (see **Figure 4**). PDAC tumor morphology is unique with normal heterogeneous features which have characteristics opposite to that of liver tumor morphology (4, 6, 19, 21). This is an important result due to the application of this equation showing a distinct contrast between the healthy pancreas and tumor.

**Figure 5:**
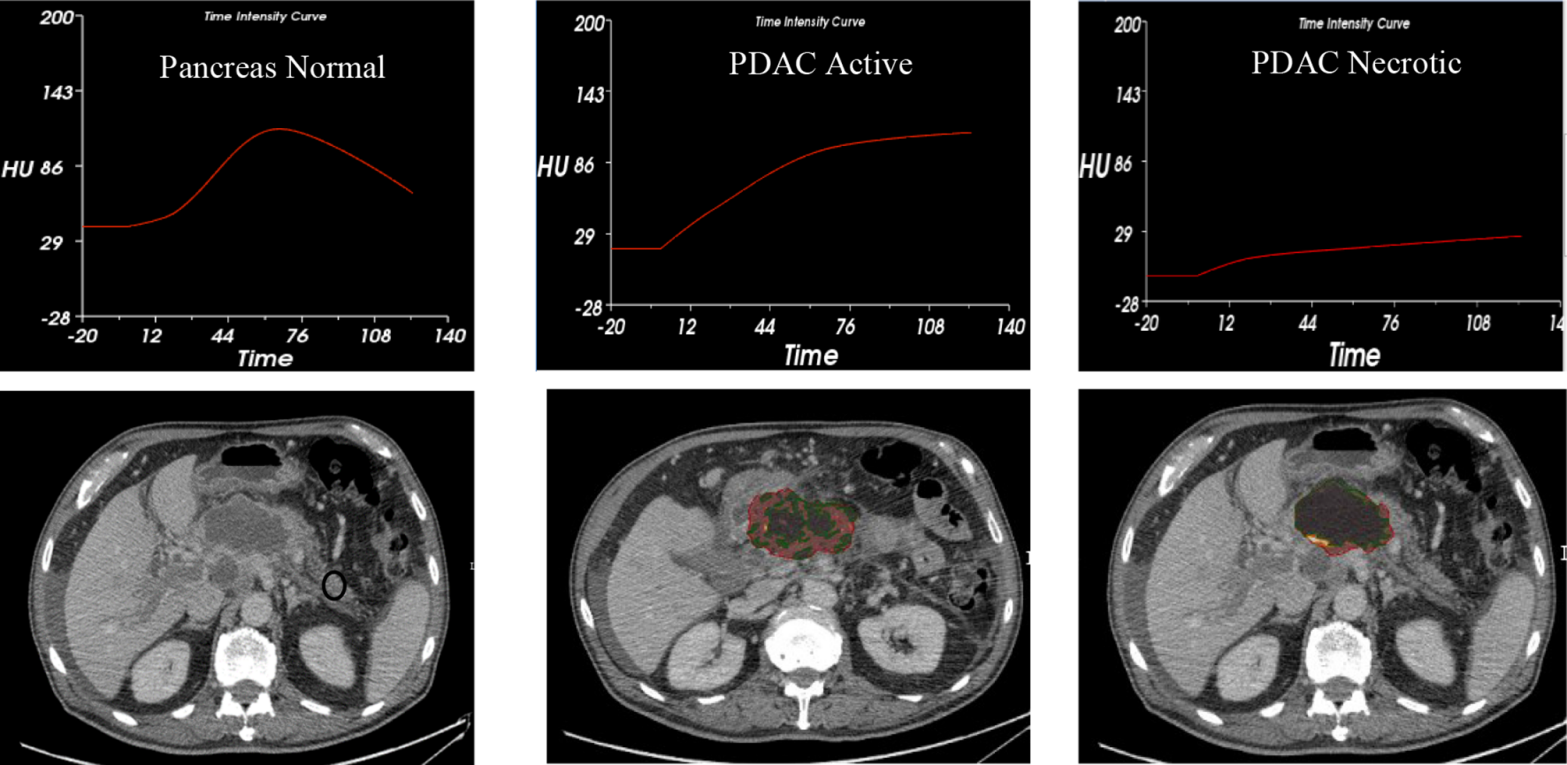
Time Intensity/Density Curves.

TDIC analysis was performed on all patient images due to the limited availability of the multiphase standard imaging protocol. This was caused by missing scans including non-contrast images or series of the arterial or venous phases. However, 3D volumetric quantification was conducted on all images. Based on the pilot data, there are indications that perfusion in PDAC exhibits delays, showing a later response, as compared to a normal pancreatic tissue (see **Figure 5**). TIDC analysis shows that perfusion takes a longer time in the venous phase in PDAC imaging. These perfusion delays represent the active form of the tumor which might be significantly associated with tumor aggressiveness or VEGF-related activity. Longer perfusion time in the venous phase was directly correlated with the aggressiveness of the disease. This groundbreaking finding shows that TIDC analyses can observe PDAC in the venous phase manifesting in a manner opposite the normal pancreatic morphological TIDC which cleared the dynamic contrast in the late venous phase.

### 3D Volumetric Analysis

**Figure 6:**
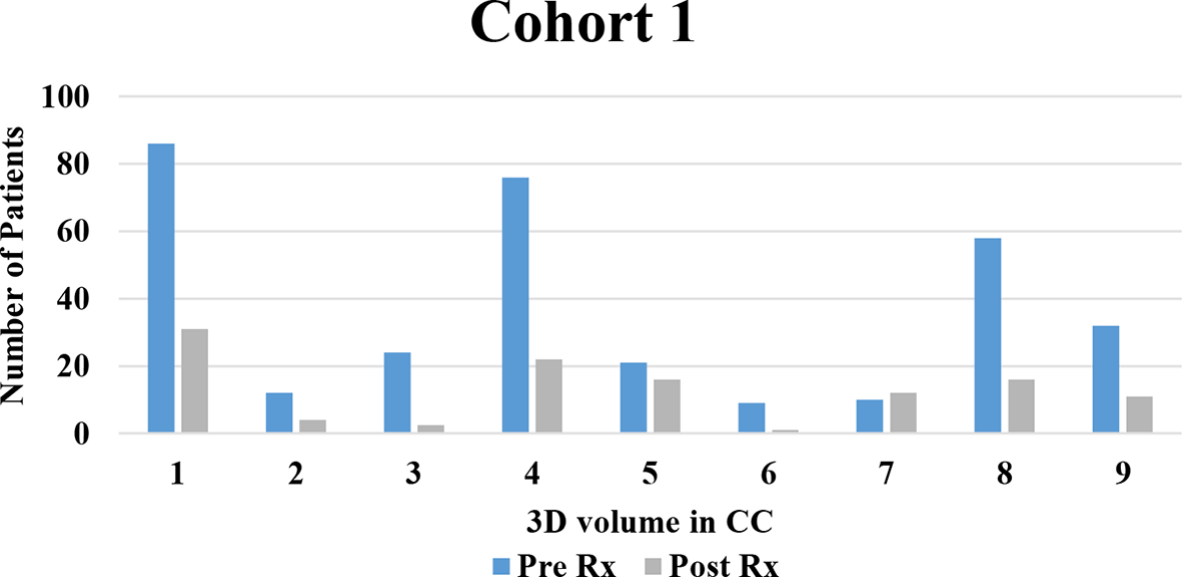
Decreases in PDAC Volume.

3D tumor volume was measured in patients diagnosed with PDAC receiving standard treatment with or without neoadjuvant therapy. A correlation of disease burden decreasing with clinical treatment was seen predominantly in Cohort 1. Changes in arterial and venous perfusion were related to tumor aggression. These findings have the potential for integration to create new screening tools which may serve as potential imaging biomarkers for the early detection of PDAC. Due to the small sample size, we were unable to produce statistical analyses, but the hypothesis is completely based on the perfusion characteristics on the follow-up imaging. Based on the findings, the PDAC volume measure decreases constantly over time which is seen with eight of the patients in Cohort 1 (see **Figure 6**), which indicates that the neoadjuvant population had a better control on the disease compared to the SOC of Cohort 2. The graphs indicated that 3D volume measurements might be a potential imaging biomarker to identify the disease progression or regression status. 3D volumetrics can provide more precise pixels based on the quantitative assessment on follow-up imaging for the those previously diagnosed with PDAC.

**Figure 7:**
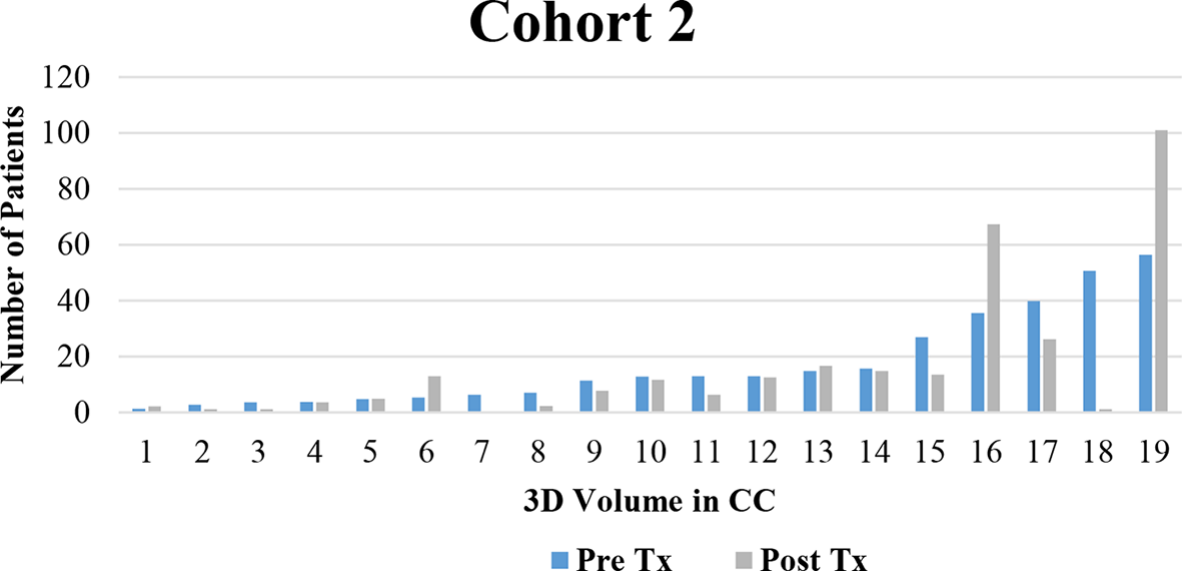
Change in 3D volume which decreased over time.

The average percent change in volume based on the images analyzed was explored (see **Figure 7**). Serial imaging in Cohort 1, who were receiving neoadjuvant therapy in addition to SOC treatment, showed steady decreases in tumor volume and venous vasculature. Imaging from Cohort 2, who were receiving standard therapy alone, displayed variable responses during treatment with an average decrease overall. This change does not display major differences compared to Cohort 1 but shows some change on the follow-up imaging. 3D volume could be a main indicator for the major and minor volumetric change compared to that of 2D analysis.

## Discussion

Pancreatic ductal adenocarcinoma (PDAC) accounts for over 85% of pancreatic malignancies (1). Despite the recent improvements to diagnostic imaging, conventional treatments, and newer targeted therapies, mortality remains high (4). The deep retroperitoneal location of the pancreas creates certain diagnostic uncertainties that limit current efforts in the early detection of pancreatic cancer (4). Multiphase CT is currently the standard modality for diagnostic testing (39–42). However, changes to tissue morphology that occur with PDAC make it difficult to define texture accurately with 2D CT imaging (39). This study evaluated the use of 3D quantitative volumetric and TIDC analysis for detecting PDAC treatment response.

**Figure 8:**
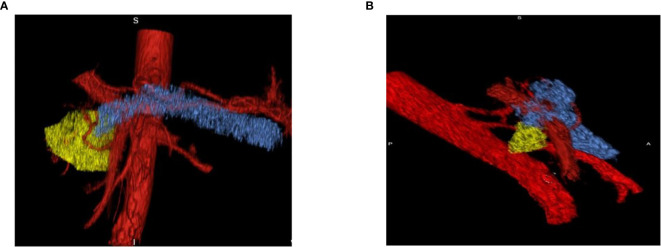
Pre- and Post-Treatment Tumor. **(A)** Pre-treatment tumor surrounded by vessels which indicates the increase in perfusion effect in tumor progression. **(B)** Post-treatment tumor volume decreases on the follow-up scan which shows that decreases in perfusion lessens the aggressiveness of the tumor.

The difference in tumor perfusion during the arterial and venous phases increased with respect to time. Results showed that volume- and perfusion-based imaging biomarkers could play a significant role in defining the aggressiveness of early-stage tumors and response to therapy. Further studies to identify additional 3D imaging biomarkers for pancreatic cancer could collectively increase the precision of early diagnostic imaging (See **Figure 8**). Based on the results, it is predicted that the longer the time spent in the venous phase might relate to the tumor aggressiveness behavior. This methodology in conjunction with the Marly Equation might be useful for the early detection of pancreatic cancer by quantifying the exact changes in the perfusion imaging. The larger the change, the more aggressive the disease. It can also be useful for comparing TIDC standard venous phase perfusion rates to PDAC cases.

Tissue radiodensity on CT images, measured in HU, changed with respect to time. Clear documentation of these changes, albeit in a small sample size, suggests that there is potential for application in the creation of a screening tool that might be used to detect PDAC; however, more information is needed. Conclusive evidence that finds this result as accurate across a larger and more diverse sample population might point toward the use of this application in diagnostic modalities. Perfusion delays represented the active form of the tumor which might be associated with VEGF-related activity. Longer perfusion time in the venous phase was directly correlated with the aggressiveness of the disease which might reflect an increased angiogenesis. In cancer, VEGF has been identified as one of the main culprits behind rapid angiogenesis which deprives healthy cells of sufficient nutrients and promotes metastases (43). Even though PDAC displays a poor perfusion, it might still have a considerable VEGF-related activity (44). Many tumors require angiogenic processes that are therapeutically targetable (45). However, this dependency indicates the demand for nutrients and oxygen exchange which is satisfied by the metabolic burden of tumor cells instead of a measure of angiogenic dependence (45). Future projects with larger patient populations might provide more insight into the relationship of PDAC aggressiveness and angiogenesis.

This was a retrospective study, which means that contact from two medical facilities from which the participant data were obtained was not standardized. As a result, several potential participants were excluded from this study due to variability in the acquisition times of images during the treatment regimens. Patients at one facility receiving a certain type of treatment may have images taken within a particular time frame after receiving treatment, while others may have images taken within a different time frame. This may cause imaging inconsistencies across the patient data that might not have been clearly documented, making it difficult to identify confounding variables and analysis less effective.

## Conclusion

The clinical application of TIDC analysis is an emerging area of perfusion imaging. Previous studies have demonstrated the utility of TIDC analysis in differentiating pancreatic solid lesions using contrast-enhanced endoscopic ultrasound. However, technician-dependent ultrasound-based techniques have been shown, historically, to be difficult to standardize and can have limited accuracy. Variations in the way technicians complete their assigned imaging tasks can result in slightly different images. Similarly, differences in available imaging technologies within treatment facilities contribute to further variations in standardization. However, increased pushes for the integration of EBP within medical screening and diagnostic modalities could suggest that future efforts in this area could be better received, with a greater potential for application. Creating a unified standard set of protocols and procedures to assess multiphase CT scans is needed, regardless of instrumentation. Focus on the types of content to be included in the images, and the different views of the area should be increased in current screening methods. This information can then be translated into different diagnostic methods that can improve the effectiveness of early detection practices; however, the integration of EBP is only the first step.

TIDC analysis in six patients demonstrated a late venous perfusion delay, as compared to perfusion rates for a normal pancreas. It was hypothesized that the perfusion patterns in the late venous phase could be used to define the aggressiveness of pancreatic cancer. Our research was limited by the small sample size of the study. It was anticipated to collect and analyze data from all patient files; however, there was only enough uniformity in the imaging practices and time frames of six participants. This limitation is brought by a lack of imaging protocols with sufficient acquisition times suggesting an area in need for improvement. Several of these concerns can be decreased and possibly mitigated entirely with the integration of clear EBPs. The construction of TIDCs requires the availability of images taken from multiple phases over a longer period of time than that which is utilized most often in standard pancreatic imaging protocols. Among the study images obtained, the peak or maximum value of the curve was not visualized for pancreatic cancer images which suggests the need for extended acquisition times. It was suggested that the time-to-peak on TIDCs might provide important information about tumor aggressiveness. It might be helpful to define the precision-based imaging findings for the detection of the PDAC. Mostly patients who are diagnosed with cancer go the SOC protocol which provides the EBP clinical information.

Our preliminary results can be beneficial in acquiring more precise information for the tumor behavior using the perfusion imaging. Projects are currently underway that optimize imaging protocols for TIDC analysis in pancreatic cancer. With this study using a smaller population, we plan to take these new imaging techniques to a larger and more diverse population to test TIDC. Through the proliferation of research in this area, the creation of an early screening tool for pancreatic cancer becomes even more likely. Rahmanuddin lab is currently working on the discovery of several imaging biomarkers based on the perfusion imaging for PDAC.

Pancreatic cancer continues to have a high mortality due to the late detection and lack of effective screening methods. The novel radiomic biomarkers described here can be applied towards detecting variations in pancreatic cancer. A potential future screening tool for pancreatic cancer can involve a combination of standard imaging protocols, quantitative imaging biomarkers, and targeted molecular assays that allow the earlier detection of pancreatic cancer in high-risk patient populations. The completion of this study, combined with the efforts of other researchers in this and similar areas of research, serves as the first step in the creation of a screening tool of this nature. Using a smaller sample size, the potential effectiveness of TIDC in applications for screening was demonstrated.

## Data Availability Statement

The raw data supporting the conclusions of this article will be made available by the authors, without undue reservation.

## Author Contributions

All authors were involved in study design, manuscript writing, collection, and the final submission process. All authors contributed to the article and approved the submitted version.

## Conflict of Interest

The authors declare that the research was conducted in the absence of any commercial or financial relationships that could be construed as a potential conflict of interest.

## Publisher’s Note

All claims expressed in this article are solely those of the authors and do not necessarily represent those of their affiliated organizations, or those of the publisher, the editors and the reviewers. Any product that may be evaluated in this article, or claim that may be made by its manufacturer, is not guaranteed or endorsed by the publisher.
